# Pet owners’ and veterinarians’ perceptions of information exchange and clinical decision-making in companion animal practice

**DOI:** 10.1371/journal.pone.0245632

**Published:** 2021-02-01

**Authors:** Natasha Janke, Jason B. Coe, Theresa M. Bernardo, Cate E. Dewey, Elizabeth A. Stone

**Affiliations:** 1 Department of Population Medicine, Ontario Veterinary College, University of Guelph, Guelph, Ontario, Canada; 2 Department of Clinical Studies, Ontario Veterinary College, University of Guelph, Guelph, Ontario, Canada; University of Lincoln, UNITED KINGDOM

## Abstract

One of the most complex aspects of the veterinarian-client-patient interaction is the clinical decision-making process. Research suggests that the approach to communication used by veterinarians can impact veterinary clients’ involvement in the decision-making process and their ultimate satisfaction. Using different approaches to the decision-making process may affect how information is exchanged and consequently how decisions are made. The objective of this study was to determine pet owners’ expectations with respect to information exchange and decision-making during veterinarian-client-patient interactions and to compare veterinarians’ perceptions of those expectations and the challenges they face in meeting them. Five pet owner focus groups (27 owners) and three veterinarian focus groups (24 veterinarians) were conducted with standardized open-ended questions and follow-up probes. Thematic analysis of the transcribed data was conducted to identify trends and patterns that emerged during the focus groups. Three pet owner-based themes were identified: 1) understanding the client; 2) providing information suitable for the client; and 3) decision-making. In addition, three barriers for veterinarians affecting information exchange and decision-making were identified: 1) time constraints; 2) involvement of multiple clients; and 3) language barriers. Results suggest that pet owners expect to be supported by their veterinarian to make informed decisions by understanding the client’s current knowledge, tailoring information and educating clients about their options. Breakdowns in the information exchange process can impact pet owners’ perceptions of veterinarians’ motivations. Pet owners’ emphasis on partnership suggests that a collaborative approach between veterinarians and clients may improve client satisfaction.

## Introduction

A veterinarian’s approach to communicating with their client has been found to impact a number of important outcomes of veterinary care including client adherence [1], client satisfaction [2], veterinarian satisfaction [3], and client recall of information [4], as well as appointment efficiency and accuracy [5]. The style of communication used by veterinarians while interacting with clients can affect the way clients participate in decision-making [6]. While various terms are often used, there are three primary approaches that veterinarians and clients can take during medical decision-making, as described by Charles [7]: paternalistic, shared, and informed. In the paternalistic role, veterinarians make decisions for their clients based on their expertise of what they medically believe would be the ‘best’ option and where communication is typically one-directional because the veterinarian assumes the client shares the same values. In the shared role, the veterinarian seeks out and provides information based on the clients’ stated preferences and decisions are made together. In this role, there is no dominance within the conversation as the client and veterinarian provide equal input. In the informed role, the veterinarian provides information requested by the client to make a decision. In this role, clients make decisions autonomously, without receiving direction or advice from the veterinarian.

While the paternalistic, shared, and informed roles were developed to describe physician-patient interactions [7], they extend to veterinarian-client-patient interactions as well [8]. Research examining the communication styles used in veterinarian-client-patient interactions have found that veterinarians historically have used predominantly paternalistic communication patterns [6,9]. While it may seem inherent for veterinarians to use a single type of communication pattern during their consultations, Shaw [9], found that companion-animal veterinarians’ communication patterns varied from interaction to interaction. Recently, it has been recommended that veterinarians employ more collaborative approaches with clients, including greater partnership and client empowerment [10]. Bard [6] suggests that veterinarians may improve outcomes relevant to veterinary care by taking a more relationship-centered approach to communication with their clients.

Studies examining pet owner expectations in veterinary medicine have been predominantly focused on general expectations related to veterinarian-client communication [11,12], end of life care [12,13], and monetary aspects of veterinary medicine [14]. Christiansen [13] investigated end of life decisions and found that some pet owners do not want to make autonomous decisions. They would prefer to have input from their veterinarian because the responsibility of making end of life decisions on their own can create too much pressure [13]. Despite differing topics, multiple researchers who have qualitatively explored pet owner expectations of their interactions with a veterinarian have found that expectations related to decision-making emerged as prominent themes or subthemes [11,15]. While these studies [11,15] suggest clients want to be involved in decision-making regarding their pet’s healthcare, to the authors’ knowledge, there have been no qualitative studies specifically examining the expectations of veterinary clients in regards to information exchange and clinical decision-making.

The purpose of this study was to compare veterinarians’ and pet owners’ perceptions of veterinary clients’ expectations with respect to information exchange and decision-making during veterinarian-client-patient interactions. A secondary objective of this study was to identify common barriers and challenges that veterinarians experience related to information sharing and decision-making.

## Methods

The study protocol was approved by the University of Guelph Research Ethics Board (REB#19-05-003).

### Focus group development

The first author (NJ) organized and conducted all data collection and analysis for this study. The only prior contact made between participants and researchers was during the recruitment process. Participants were made aware that the research was being conducted for the first author’s (NJ) PhD and of the broad objectives of the study. The focus group discussion guides were developed by the research team. Three pet owner pilot focus groups and one veterinarian pilot focus group were held at the Ontario Veterinary College to assess the study protocol, to familiarize the moderator with the discussion guide and to obtain feedback from participants on the clarity of the questions.

### Study design

Five pet owner focus groups and three veterinarian focus groups were held between July and October 2019. Each focus group involved 2–9 participants, one moderator (NJ) and one research assistant. The research assistant was present to take notes of prominent topics within each session. Three 2-hour pet owner focus groups were held in a boardroom at the Ontario Veterinary College in Guelph, Ontario, Canada and two were held in a private room within a public library in Kitchener, Ontario, Canada. Veterinarian focus group sessions were held for 2.5 hours (including a 1-hour dinner); one of which was held in a hotel conference room in Guelph, Ontario, Canada and two were held in a private room within a restaurant in Kitchener, Ontario, Canada. A semi-structured interview format was used to conduct pet owner and veterinarian focus groups, which included a set of open-ended questions and follow-up probes, found in Table 1. Member checking, a method in which data and interpretation of data is reviewed with participants [16], was implemented at the end of each focus group. Raw data captured by the research assistant throughout each focus group were reviewed with participants to ensure the accuracy of information captured. All focus groups sessions were audio recorded using standard recording equipment (Zoom H2n Handy Recorder, Zoom North America, Hauppauge, New York) and transcribed verbatim by a professional transcriptionist external to the research team.

**Table 1:** Discussion guide questions for pet owner and veterinarian focus groups.

**Pet owner discussion guide questions**
Think about a positive experience you’ve had at the veterinarian, what contributed to this experience being a positive one? Think about an experience that was less than ideal, what contributed to this experience being less than ideal? Thank about the ways that veterinarians have presented information to you in the past, what made sharing information with you effective? What was not effective about the way veterinarians have shared information with you in the past? How does the effectiveness of the way your veterinarian shares information with you impact the way you make decisions about your pet’s health?
**Veterinarian discussion guide questions**
What do you perceive creates a positive experience for your clients? What are some of the challenges that you experience day-to-day with your clients? How do you most effectively share information with your clients? What are the challenges that you face as veterinarians when sharing information with your clients? How does the effectiveness of the way you share information with your clients impact the way they make decisions about their pets’ health?

### Participants

Client participants were recruited from veterinary clinic lobbies and from posters promoting the study in veterinary clinics located within a 30-minute drive from the Ontario Veterinary College in Guelph, Ontario, Canada during July and August 2019. Purposive sampling [17] was used to select 20 veterinary clinics for client recruitment. In order to obtain clients from different practice types, 10 clinics were chosen that had three or fewer practicing veterinarians and ten clinics where chosen that had greater than three practicing veterinarians (range from 4–12). Clinics were contacted to ask permission to recruit a convenience sample of companion animal pet owners from their clinic lobby and to place a poster in their clinic to promote the study. Five clinics agreed to in-person recruitment and twelve agreed to display a poster promoting the study within the lobby of their clinic. For each clinic that was in agreement to in-person recruitment, the principal author (NJ) conducted in-person recruitment for two days. All pet owners who agreed to participate in the study selected one out of five possible focus group time slots. Participants were contacted via email or phone one week and 24 hours prior to their designated focus group time with a reminder. At the start of each focus group session, consent was reviewed by the moderator and each participant provided written consent and completed a demographic questionnaire. Pet owner demographic information collected included: gender, years of experience taking their pet to any veterinarian, average number of routine veterinary visits per year, and number and type of pets. Client participants were provided with refreshments during the focus group and a $25 honorarium at the end for participation. Inclusion criteria included that participants had to be at least 18 years of age, English speaking, the primary care giver of a dog or cat, and had visited the veterinarian with their pet in the last year.

A randomized list of veterinarians who practiced within a 30-minute drive of the Ontario Veterinary College was obtained from a publicly available database on the College of Veterinarians of Ontario’s website (www.cvo.org). Recruitment of veterinarians was conducted during September and October 2019. Randomly selected veterinarians were sent an email invitation to participate in the study, followed by a phone call from a member of the research team within one week. Veterinarians who expressed interest in participating were provided with a selection of three dates and subsequently signed up for one focus group session. Veterinarians were contacted via email with a reminder one week and again 24 hours prior to their focus group session. Consent was reviewed by the moderator and each participant provided written consent. Participants completed a demographic questionnaire at the start of each focus group session. Veterinarian demographic information collected included: gender, years in practice, hours worked per week, time spent practicing small animal medicine, and role at the practice. Veterinarian participants were provided with dinner and a $50 honorarium at the end of the focus group session. To be eligible to participate, veterinarians were required to be English speaking and practice at least 50% companion animal medicine at the time of the focus group. Veterinarians who practiced in mobile practices and emergency-only clinics were excluded.

Demographic data were summarized to describe the participants involved in both pet owner and veterinarian focus groups. While purposive sampling of pet owners enabled the recruitment of pet owners from a variety of clinics and random sampling of veterinarians facilitated the recruitment of a representative sample of companion animal practitioners, caution should be taken in generalizing results to a wider population due to the qualitative nature of this study.

#### Thematic analysis

Verbatim transcripts were reviewed, in conjunction with listening to the audio recordings, by the principal author (NJ) to ensure the accuracy and quality of the transcripts. Thematic analysis [18] was performed first on the transcripts from the pet owner focus group using standard software (Quirkos 2.3, 2020). Initial codes were developed using an inductive coding approach, a process in which the codes and themes develop from the data, rather than following a pre-existing theoretical framework [19]. Transcripts were reviewed for reoccurring matters that arose throughout the focus groups. Sections of transcripts were reviewed, and interpretations of codes were discussed between the first and second author (NJ and JBC) to confirm agreement on interpretations being made, a validation method known as peer debriefing [16]. Codes were subsequently used to inform the development of subthemes and themes, through an iterative, multi-step process. Codes that were emergent in the pet owner focus groups were then used to deductively analyze the veterinarian focus groups to allow for comparison of thematic content. An inductive approach was also used to analyze veterinarian focus groups to explore veterinarian-specific barriers related to information exchange and decision-making.

## Results

### Participants

A total of 27 pet owners participated in 5 focus groups. All pet owner participants owned at least one dog or cat, and all pet owners visited the veterinarian at least once per year, with 89% (24/27) visiting the veterinarian three or more times per year.

A total of 24 veterinarians participated in 3 focus groups, all of whom were strictly small animal practitioners. Two participating veterinarians practiced at the same clinic and participated in the same focus group, while the remainder were all recruited from separate clinics. The median number of veterinarians that practiced at the clinic of participating veterinarians was 3 (mean 4.3), ranging from 1–19. See Table 2 for additional pet owner and veterinarian participant demographics.

**Table 2:** Demographic characteristics of participating pet owners and veterinarians.

Pet Owner Participants (n = 27)	n (%)	Median, Min-Max
Gender		
Female	24 (89)	
Male	3 (11)	
Number of pets owned		2, 1–12
Number of years visiting any veterinarian		17.5, 1.5–47
**Veterinarian Participants (n = 24)**		
Gender		
Female	22 (92)	
Male	2 (8)	
Role at clinic		
Practice owner/partner	12 (50)	
Associate/locum	12 (50)	
Hours worked		
Part-time	12 (50)	
Full-time	12 (50)	
Years since graduation		14.5, 2–41

**Fig 1:**
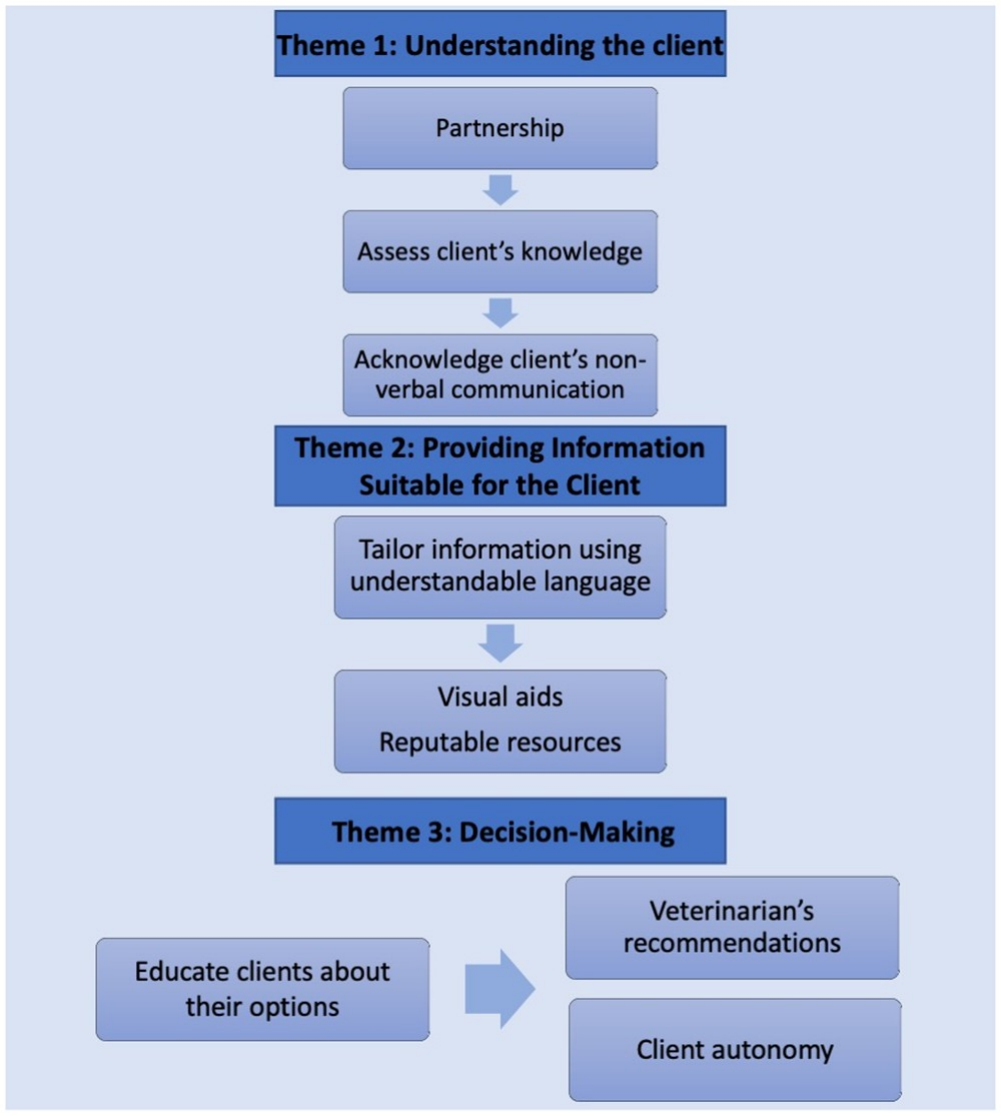
A concept map of the themes and subthemes related to information exchange and decision-making expectations that emerged from pet owner focus groups. Concepts move from top to bottom and are presented in the order in which they may logically flow within a consultation.

Thematic analysis of the pet owner focus groups revealed 9 overarching themes: continuity of care, decision-making, end of life care, monetary aspects of veterinary care, physical environment of the clinic, providing information suitable for the client, understanding the client, veterinarian-client-patient relationship, and veterinary support staff-client-pet interactions. The current study reports on the three themes related to information exchange and decision-making, which are specific to the objectives of this study. The three themes presented herein include: 1) understanding the client; 2) providing information suitable for the client; and 3) decision-making. Inductive analysis of the veterinarian focus groups also identified veterinarian-specific barriers that impede information exchange and decision-making, including time constraints, involvement of multiple clients and language barriers. A concept map of themes and subthemes that emerged from pet owner focus groups is presented in Fig 1.

### Theme 1: Understanding the client

#### “It’s a partnership”

Participating pet owners expressed the expectation of being *“a team”* or *“in partnership with [their veterinarian]”*. A good partnership between a veterinarian and a client was described as two or more individuals who have learned to successfully exchange important information back and forth relevant to providing care and making decisions for a patient. Participants explained that partnering with a veterinarian had a positive impact on their relationship; one pet owner talked about *“how important it is or how good it feels to be included as a partner in the decision making”*.

Pet owners in four focus groups emphasized that an important aspect of partnership involves the veterinarian respecting an owner’s knowledge and understanding of their own pets because they are the ones who *“speak for their pet”*. Pet owners expressed the need for this partnership to begin with the veterinarian understanding the clients’ observations and concerns. When a veterinarian does not acknowledge their concerns, observations, or sense of urgency, participating pet owners described often being left with a feeling of uncertainty and felt that the veterinarian ended up *“rushing to conclusions and not really being aware of my concerns”*. Participants articulated that their veterinarian’s medical knowledge should be combined with the participant’s observations and understanding of their pet in order to fully understand the next steps that need to be taken. As one participant commented: “*Because my expertise is the knowledge of the animal and observations and then the veterinarian brings a lot of professional knowledge to the table but if you’ve built the information sharing relationship*, *that is very powerful*.”

Only two veterinarian participants specifically mentioned being *“partners”* and being *“team based”* with their clients, veterinarians in the other two focus groups discussed how listening to clients’ observations, while gathering history, positively impacted veterinarian-client rapport by allowing clients to become more engaged. Gathering history was expressed as a challenge by veterinarians in two focus groups, as some participants felt that their clients were not engaged in their pet’s veterinary care because the clients did not communicate their concerns or observations. Some veterinarians emphasized the importance of being *“on the same page”* as their clients, however, a few other veterinarians found this to be a challenge when they had different priorities than their clients. As one veterinarian remarked *“I have a priority and the client has a different one so getting those to mesh are hard sometimes*.*”* This was most often discussed as a challenge when the client wanted to discuss less urgent health matters and disregarded their veterinarian when they raised new or what they believed were more urgent concerns.

#### “Just listening” and “Being aware of my concerns”

Pet owners in four focus groups discussed at length the importance of veterinarians demonstrating that they were listening, by acknowledging clients’ thoughts and opinions, which included both listening to what clients say verbally and by evaluating body language to assess clients’ emotional state. Participating pet owners expressed that listening requires veterinarians to focus on the present moment, *“being mindful of the person and animal standing there”*, as this is necessary to be able relate to their clients. As one pet owner recalled an experience when their emotional cues were being ignored, *“I was visibly upset and the vet kind of didn’t really acknowledge that*.*”*

The majority of participating veterinarians mentioned listening to clients when initiating appointments as an important contribution to a positive client experience. Listening to clients’ concerns was raised by veterinarians as a mechanism to engage clients and build rapport while minimizing assumptions. A few participating veterinarians recognized the importance of acknowledging clients’ emotions, yet many expressed challenges around reading clients’ body language, which often contributed to uncertainty over understanding their clients. Some veterinarian participants expressed difficulty *“being present”* with clients while having other concerns on their mind or when feeling emotionally drained.

#### “Reading where you are at with your knowledge base”

The majority of pet owner participants raised the importance of the veterinarian recognizing the level of information sought by each individual client. Participating pet owners expressed appreciation for when their veterinarian assessed the client’s current knowledge base or their desire for information upfront, in order to vary the amount and way information is conveyed. Participants viewed it as being supportive and expressed that their veterinarian was treating them as an equal partner in the care of their pet. Ensuring clients had all of the relevant information for them to make informed decisions allowed clients to feel that they were able to provide the best care they could for their pet. As one pet owner recalled, *“She understood that I was there for information and I was very bonded to this dog*, *and I wanted to know the information that I needed to be the best custodian to this dog as possible”*. Despite varying degrees of information needs, many participating pet owners expressed that more information is better than receiving a vague explanation, even if they do not understand everything, as this provides them with the opportunity to ask questions: *“I want to know everything even if I don’t understand it because then I can just ask about it rather than not having it at all”*.

Many participants in the veterinarian focus groups felt that knowing their clients was the first step to being able to communicate with them effectively, as every client can have different preferences. Participating veterinarians acknowledged the importance of adapting explanations to the level of understanding of each individual client yet highlighted having difficulty *“explaining complicated issues in a way that [clients] can actually understand… Even simple concepts sometimes”*. The majority of veterinarians focused on checking in with clients during discussions by asking if clients have any questions and reading body language to check understanding, with minimal emphasis on how they assessed clients’ current knowledge prior to providing explanations.

### Theme 2: Providing information suitable for the client

#### “Explain it to me in terms that I can understand”

Participants in all pet owner focus groups agreed that they wanted their veterinarian to explain things in a way that they understood. As one pet owner said: *“I don’t have a medical degree and I have no intentions of getting one*, *just because I own an animal*, *so explain it to me in terms that I can understand*”. Some pet owners indicated that when their veterinarian used medical jargon they *“have to go home and Google what they’re saying”*. Though preferences for use of language varied across participants, they expressed that it was important for veterinarians to tailor the language they used to the individual client as some participants associated this with a display of respect. As one pet owner stated, *“I respected the fact that she knew I was well informed*, and another said, *“she knows that I understand at a certain level…she doesn’t dumb things down”*.

Aside from one participating veterinarian who mentioned that they have difficulty removing medical jargon during discussions with their clients, the veterinarian focus groups did not discuss tailoring their language or avoiding medical jargon.

#### “Visual aids…coupled with the verbal explanation is often very, very helpful”

Participating pet owners in three focus groups discussed their difficulty comprehending new information when it was only conveyed verbally. Some of these pet owners explained that if veterinarians rely on verbal explanations, they should be able to explain things in a variety of ways, rather than repeating the exact same explanation. Participating pet owners across four of the focus groups mentioned that they found using a visual aid alongside a verbal explanation was an effective method for their veterinarian to share information with them. Participants described using *“pictures from textbooks”*, *“the internet”*, *“x-rays”*, or visually *“demonstrating on the animal”*, to explain a diagnosis as especially helpful when comprehending new information. One client explained, “*And some of it was all new to me*, *so she had pictures*. *You know like to hold up and say this is that and this is how this works*, *and I understood it completely*, *between the words and the pictures I knew what she was talking about*.” After hearing other pet owners’ experiences, a number of pet owners who mentioned only having ever received information verbally from their veterinarian, expressed an interest in having information communicated in multiple ways by their veterinarian.

In all veterinarian focus groups, participants briefly mentioned using visual aids, such as *“pictures”*, *“models”*, *“diagrams”*, *“x-rays”*, *“hand-drawings”*, *and “demonstrating on the animal”* to support their explanations, “*because I think that sticks more than me just yammering on over things*.*”* Veterinarians in two focus groups discussed methods used to verbally convey information to their clients to enhance a client’s recall of information, specifically, repetition and analogies being the main techniques discussed. A few veterinarians mentioned their team was also important for ensuring information was repeated to their clients. One veterinarian described: *“just the plain volume of information that we want to transmit*, *and people don’t remember it*, *so you have to do something else to jog their memory*, *otherwise they phone up the next day and they did something completely different than what you suggested”*.

#### “Having a handout would honestly be really, really helpful”

Pet owners in four focus groups conveyed appreciation for receiving informational handouts from their veterinarian for them to review on their own time because it allowed them to reflect on what was discussed during their appointment. This was especially important during times of emotional distress for a client, for example, when receiving information about a new diagnosis or when clients witnessed their pets in distress, as some participants felt that these scenarios made it difficult to absorb information. Pet owners did not expect their veterinarian to discuss the information they provided in handouts in detail yet expressed not wanting to *“feel bad”* for following up with their veterinarian if they needed to after an appointment. Participants expressed that follow-up from veterinarians gave them the opportunity to clarify information or express concerns following their appointment. As one client recalled: “I *was so traumatized because my cat was crying*. *My cat never cries*. *To retain some of the information she gave me it was just like too much…I need to talk to you a couple days later when I’ve calmed down*.” One client shared an experience of getting so many handouts that the crucial information was lost in the number of handouts they received.

Veterinarians in all focus groups felt providing additional resources for clients was important because clients may otherwise forget what was discussed. Although participating veterinarians widely discussed using handouts, some doubted whether their clients read them. One veterinarian stated, *“I never give a handout without explaining what’s on it because I don’t trust that people will read it*.*”* While veterinarian participants felt that handouts provided clients with something tangible to take home, adding value to their service, some mentioned discontinuing the use of personalized handouts due to the time associated with preparing them. Veterinarian participants also described clients’ emotions as a challenge because emotions often interfered with the clients’ ability to absorb information. Handouts and following up with clients were two methods that veterinarians used to ensure they were on the same page as their clients after appointments, though many veterinarian participants described difficulty finding time to follow-up with clients after providing information.

#### “I go home and I Google right away”

Pet owners in all focus groups mentioned searching the internet in some capacity, whether they checked *“Google”*, *“Wikipedia”*, *“Blogs”* or *“Chat Rooms”*, yet many acknowledged that these sources are not as reputable as sources of information provided by their veterinarian. Pet owners discussed searching the internet due to a desire for more pet health information, however, they also expressed wanting their veterinarian to provide reputable sources of information: *“I looked it up*, *on Wikipedia*, *but I wanted something professional”*. Another pet owner recalled, *“It would be nice if they had a resource tool you know*, *like a little website that you could go to*, *instead of Googling it and getting 15 different answers*.*”* While the majority of pet owners mentioned searching the internet after visiting their veterinarian, some pet owners discussed booking appointments with their veterinarian after reading information online, or in the media, in order to have their veterinarian clarify what information was reliable and to help them “*make an informed decision*.” A few client participants mentioned not feeling the need to seek additional information, as they trusted their veterinarian to provide the information they needed and make the best recommendations for them.

Veterinarian participants throughout all veterinarian focus groups discussed clients seeking alternative information sources broadly and primarily recognized it as a challenge. Several veterinarians in each focus group felt that clients prioritized pet health information received from other sources over information from their veterinarian, leading to decreased client engagement with their veterinarians. This trust in non-veterinary sources, including pet store employees, breeders, and the internet, was a particular challenge in relation to clients seeking information about nutrition. The internet was the most widely discussed challenge in relation to client information seeking behaviours, as one veterinarian participant expressed, *“Oh Google*, *it’s ruining our lives”*. Veterinarians did convey that the majority of their clients understood that information found on the internet may not always be reputable. Only two veterinarians expressed that clients seeking information on the internet was an opportunity for a discussion about what the client found online and the participants felt it showed that the client was engaged in their pet’s healthcare. Some participating veterinarians also mentioned the importance of providing *“good”* and *“reputable”* websites for clients, knowing the clients would likely search the internet.

### Theme 3: Decision-making

#### “Explaining the options” and “Having the final say”

Being given options was discussed in breadth and depth in all pet owner focus groups. Participants expressed interest in being well-informed about their options and appreciation for veterinarians who understood that they wanted to make informed decisions and do what is best for their pet. Participating pet owners discussed the importance of being told the necessity of diagnostic or treatment options as this helped them understand the long-term effects of their decisions. Several participants mentioned wanting financial estimates to be part of the discussion of options. The majority of participants emphasized wanting to know *“all of the complexities of how it affects my dog”*. As one pet owner described *“I really appreciate when a vet takes into account the impact of the options on the pet”*.

Pet owners across four focus groups felt that they held the responsibility of being the advocate for their pet that *“cannot speak”* for itself and expressed that being provided options gave them some reassurance that they had control over decisions related to their pet’s care. Participating pet owners indicated that they recognized they had the ultimate say in decisions related to their pet’s healthcare, as stated by one pet owner, “*because at the end of the day*, *it’s my job to make that decision as to what to do next*.” One pet owner mentioned wishing their veterinarian would *“have been more helpful in making that decision”* while reflecting on their experience involving the decision to euthanize a pet.

While discussing options, it was important to pet owners that their veterinarians were honest about their pet’s prognosis and didn’t give them *“false hope”*. It was additionally important to participants that their veterinarians were honest about any professional limitations that existed, for example, having limited experience treating specific diseases, as this often led to their veterinarian researching the latest treatment options or recommendations to a specialist. Participating pet owners often described that when veterinarians provided options, it reduced the clients’ concerns about veterinarians’ financial motivations behind their recommendations. As one client explained “*So they give you all these different options and it’s not just like they’re trying to make a sale or pushing one drug over another*. *So I like that*”.

Veterinarians in all focus groups recognized that clients wanted to be provided options, as one veterinarian stated, *“Most clients want to know more than one option”*. Veterinarian participants described two main approaches to providing options in practice. The most common approach involved providing more than one option upfront to all clients, as many of the veterinarians believed it was part of their job to come up with options and did not want their clients to feel pressured. Some expressed feeling more satisfied with their interactions when they fully informed clients of their options and thought that it helped clients understand the value of each option. As one participating veterinarian commented: “*I think when I feel like I’ve communicated effectively … and I feel like the owner really has all the information then I’m usually satisfied with the decision they made even if it’s not best medicine*.” Veterinarians who stated that they always provide their clients with options emphasized the importance of not judging their clients based on the option they chose, even if it is not *“best medicine”*, in the veterinarian’s opinion.

In the other general approach, veterinarians did not initially provide options, rather, they described providing the gold standard upfront and allowing clients to initiate conversations about options, which veterinarians described often occurred when clients were *“cost conscious”*. Veterinarians described wanting to provide all clients with the gold standard to offer all clients the same standard of care and ensure that veterinarians are *“not judging the book by its cover”*. One veterinarian explained, *“I always say I offer every single client that walks in the gold standard regardless and then we work back from there based on what they can manage”*.

Veterinarians in two focus groups specifically mentioned the necessity of each option when working with cost conscious clients and prioritizing what is necessary. Veterinarians across all focus groups mentioned the importance of providing cost estimates prior to any treatments or procedures as it may affect the client’s decisions and minimizes surprises on their bill. This was especially important when clients told them to *“do whatever you need to do”* for their pet, because veterinarians felt that clients often wanted different options once the cost estimate was provided. While veterinarians felt it was important to respect cost conscious clients, they emphasized the importance of not feeling guilty about the costs when providing estimates to these clients.

#### “Being told what to do” can be “Uncomfortable” and “Awkward”

Pet owners in all focus groups reflected on past experiences with veterinarians who did not present them with options and who appeared to make decisions on behalf of the client, without involving them in the decision. Several participants described “*being told what to do instead of being presented as an option*”, as being “*uncomfortable*” and “*awkward”*. As one client recalled “*it felt like a power struggle and there were no options that were given in that moment either*.” This approach to decision-making was thought to impede information exchange between veterinarians and clients, as clients expressed it limited their ability to communicate potentially important information. As one pet owner participant affirmed: “*So my input and your medical input*, *they need to blend together*, *you know*? *You can’t be a hammer and a nail where you kind of just want to beat me into submission into your way*. *It’s just not going to work*”, which was an experience described by numerous participants across all five focus groups. Many participants who described a situation where they felt their veterinarian limited their opportunity to choose, mentioned either leaving the practice or seeing a different veterinarian within a multi-veterinarian practice.

Many participating pet owners associated *“feeling pressured”* or a lack of involvement in the decision-making process with the belief that veterinarians’ intentions were solely based on money. As one pet owner recalled, *“So I kind of left feeling like*, *was this about money*? *Making money*?*”* Participating pet owners appeared to associate a lack of options with veterinarians’ financial motivations, most often discussed in relation to nutrition, with participants expressing they feel that the veterinarian *“pushed a certain brand of food because they get a deal off of it”*, rather than letting the client make the *“most informed decision”*.

Although some participants described negative experiences related to *“being told what to do”*, pet owner participants in three focus groups mentioned wanting to hear their veterinarian’s recommendations. As suggested by one client “*And I would always throw it back to them*, *what would you recommend if this was your dog*?*”*. Clients mentioned taking their veterinarian’s recommendations into consideration while contemplating their options. One client stated ***“****it was really important that our vets spoke to us with respect and spoke to us on our level rather than talking down to you*, *or giving you orders or—it was more of a this is the recommendations and provided informed opinions*.*”* A few pet owners who discussed being open to recommendations from their veterinarian also described a high level of trust in their veterinarian’s opinion.

Though veterinarians did not discuss making decisions on behalf of their clients, veterinarians across two focus groups expressed the desire to practice gold standard medicine which can be influenced by cost, because costs can be *“challenging to clients which is then challenging to [veterinarians] because [they] can’t practice the way that [they] want to in a lot of situations”*. Though participants wanted to practice best medicine, many recognized that clients don’t want to feel pressured by only having one choice, therefore some veterinarians described offering at least two options so that clients do not feel that *“they’re in a corner”*.

When veterinarian participants discussed their experience with the commonly asked question, *“what would you do if it were your dog*?*”*, the participants had diverse reactions. Some veterinarians did not enjoy making this type of recommendation because they either did not want to place their values on the client or they did not feel their recommendation would be feasible for the client, while others took this as an opportunity to suggest the *“gold standard”* treatment. Most veterinarians expressed only offering recommendations when asked by their clients, when clients were struggling to make a decision on their own or when they had built a trusting relationship with their clients over time. Veterinarians expressed challenges making recommendations when they felt that their clients had different priorities.

### Veterinarian-specific barriers

Time constraints were the primary concern raised by participating veterinarians. Feeling rushed but trying not to appear rushed with clients was important to veterinarians as they recognized it affected the client experience. Having standardized appointment lengths was considered challenging because of variable client needs. The majority of veterinarians expressed difficulty relaying information when multiple clients were involved in the care of the pet because it was difficult to get everyone *“on board”* to make decisions and veterinarians felt that it was extremely important for them and their clients to *“be on the same page”*. Language barriers, for example, if the client had difficulty understanding English, made it challenging for veterinarians to feel that they have properly explained information and therefore felt that clients were unable to make well-informed decisions. Some veterinarians mentioned employing staff that speak multiple languages as an aid to manage language barriers.

## Discussion

The results of this study provide an understanding of participating clients’ expectations and participating veterinarians’ perceptions of clients’ expectations with respect to information exchange and decision-making in companion animal practice. Previous qualitative research [11] documented pet owners’ expectations of involvement in decision-making, the participating veterinarians in that study expressed ambiguity concerning whether this expectation was driven by pet owners or veterinarians. While caution should be taken when generalizing results of qualitative research, the current study findings suggest that pet owners drive this expectation of partnership. Additional pet owner expectations expressed in the current study corroborate findings of previous qualitative research, including the expectation that information should be available in multiple formats, the use of understandable language, and listening to clients’ concerns [11,12]. The veterinarians’ challenges expressed in the current study also align with those expressed by veterinarians in an earlier qualitative study, despite over a decade separating these studies [11].

Results of the current study suggests that initial feelings of partnership could be established during the information gathering portion of the medical interview by listening to client concerns and respecting their knowledge of their pet. Veterinarians in the focus groups expressed the challenge of having different priorities than what they perceive as their clients’ priorities. However, a 2011 study [5] reported that veterinarians solicited clients’ concerns at the beginning of an interaction in only one-third of companion animal appointments and frequently interrupted once a client began talking. While there is often a mismatch between clients’ and veterinarians’ expectations as to what should be prioritized in a consultation [20], taking time to understand clients’ concerns initially may assist veterinarians in setting an agenda for the interaction that addresses both parties’ priorities [21]. When veterinarians did not actively solicit clients’ concerns at the beginning of an interaction, clients were four times more likely to raise concerns at the end of the appointment [5]. Thus, open-ended inquiry (e.g., “What concerns would you like to discuss today?”) can be used to actively soliciting clients’ concerns at the beginning of an appointment, which may help establish an initial feeling of partnership desired by clients and avoid late rising concerns at the end of the appointment [5].

In contrast to previous studies, pet owners in the current study discussed an expectation for veterinarians to acknowledge their clients’ body language and facial expressions, suggesting that clients are aware when their veterinarian misses the opportunity to engage with them. Non-verbal cues or indirect comments are often used in both human [22] and veterinary medicine [23] by patients and clients to provide their ideas, concerns and expectations, without vocalizing explicit concerns. When veterinarians overlook or ignore client cues [23], they miss opportunities to explore client feelings, ideas and expectations, ultimately reducing client involvement in defining the problem and creating a treatment or management plan [24]. Recognizing and acknowledging clients’ non-verbal cues by checking-in or eliciting the client’s perspective and concerns, provides veterinarians an opportunity to incorporate clients’ ideas and perspectives into the veterinarian’s explanation and planning. Though the Calgary Cambridge Guide is taught in most veterinary curricula [25] and incorporates demonstration of appropriate non-verbal behaviours, educators should also emphasize the importance of recognizing and acknowledging non-verbal behaviours of clients.

Participating pet owners described increased difficulty understanding information when it was new to them. It is critical for veterinarians to be aware of clients’ current knowledge as clients in novel situations may have even greater expectations for information [15]. This highlights the importance for veterinarians to assess the clients’ current knowledge before providing information, which can be accomplished by inquiring about the client’s current understanding of a topic (e.g., Tell me about what you already know about diabetes in dogs?). In addition, assessing the level of information desired by clients would assist veterinarians in deciding opportune times to provide clients with informational handouts, particularly as participating pet owners expressed wanting information to be tailored to their individual information needs. Participating veterinarians expressed uncertainty around whether clients utilize the information provided in handouts [11] and participating pet owners expressed the need to ask questions and clarify information received from handouts after their appointment. If veterinarians do not provide an opportunity for clients to ask questions about information received from handouts, veterinarians may assume that the information is not useful for the client, yet a lack of written information has been associated with reduced client compliance [26]. Additionally, the absence of follow-up telephone calls from veterinarians may further reduce client compliance [26]. These results emphasize the importance of providing clearly written information to clients and even more so the importance of providing a plan for following up with a client or letting clients know they can contact the veterinary team as needed.

The use of the internet by clients received a lot of attention in the present study from both the pet owner and the veterinarian participants, unsurprisingly, as the internet can be the primary source of pet health information for pet owners [27], and veterinarians have previously expressed concerns over the quality of sources that pet owners find on the internet [28]. Veterinarians in the current study believed that pet owners prioritize non-veterinary sources of information, whereas pet owners described using a variety of resources to become further informed and articulated a wish for reputable sources to be provided by their veterinarians. Research suggests most pet owners would visit a website recommended to them by their veterinarian [27], yet few are given these resources [27,29]. Additionally, when pet owners use the internet, they may be more confident in asking their veterinarian questions and feel better able to communicate with their veterinarian about their pet’s health [27]. Recognizing the internet is a source of information for pet owners, veterinary practices may want to consider engaging pet owners in conversations about what they have learned prior to the appointment and to direct clients toward reliable resources.

The current study revealed novel findings related to pet owners’ perceptions when options were not provided for them to make informed decisions related to their pet’s health. Previously, pet owners’ expressed expectations of being provided with a range of options [11,15]. Pet owners in this study further elaborated that when only one option was provided, they were more likely to feel that their veterinarian’s recommendation was financially motivated. In actual fact, these types of poor communication practices may have negative economic implications for clinics [29], as many pet owners in the present study described situations where they changed veterinarians or veterinary clinics after being treated with paternalistic approaches. Pet owners emphasized that a paternalistic approach to communication by their veterinarian left them with negative feelings. Nevertheless, paternalistic communication patterns remain prevalent in veterinarian-client interactions [6,9], with one study [30] finding only a small portion of veterinarians’ communication dedicated to client activation and partnership. When describing veterinarians taking a paternalistic approach, participants frequently referred to conversations about nutrition and often questioned the veterinarian’s motivations around selling pet food. A previous study [31] found that first year veterinary students and pre-veterinary students likewise feel conflicted regarding veterinarians’ motivations behind their recommendations for pet food. The current results support suggestions for veterinarians to practice communication strategies aligned with shared decision-making when addressing dietary changes [32,33]. It is recommended that clients’ preferences related to dietary changes are sought [32], while informing clients of the health issues that these changes will address and educating clients about their options [33]. By describing dietary options on a higher order than the brand or product names, there is potential for clients’ suspicion of veterinarians’ financial motivations to be reduced [33]. The present study suggests that using a shared decision-making approach may reduce the mistrust that clients experience compared to a paternalistic approach, however, this was not specifically studied.

Many veterinarians in the current study described providing more than one option for their clients, rather than only the gold standard, to ensure that clients do not feel pressured. This approach was more prevalent in comparison to a study [11] which found that only some veterinarians specified providing options and some did not believe their clients wanted options to be provided. Some participating veterinarians expressed the need to provide options only when the cost of the gold standard care was not feasible to the client, implying they rely on the client to initiate a conversation about options. Veterinarian participants suggested this approach decreased any preconceived notions of what each client can afford and avoided prejudice. Moreover, it is possible that this approach is driven by a desire to practice best medicine and undermines clients’ wishes to be provided with options upfront. In contrast to some participating veterinarians’ emphasis on cost when providing options, participating pet owners suggested multiple options should be provided upfront with cost as one of several aspects of discussing options, along with the benefits and reasons for each option in relation to their pet’s health. A more collaborative approach between veterinarians and their clients may increase veterinarian and client satisfaction [3,34]. Veterinarians should consider offering options upfront to all clients, as they may be more prepared to follow veterinarians’ recommendations [1,35].

Although pet owners have previously [15] expressed the expectation to receive sufficient information to make an informed decision, signaling a desire for autonomy, this may change depending on the weight of the decision [13]. Participating pet owners also expressed value in receiving their veterinarian’s recommendation when discussing options, which reflects the opportunity for veterinarians to take a shared role in decision-making. A shared decision-making model involves a two-way exchange of information, where both the veterinarian and the client provide input throughout all stages of the decision-making process [7]. When involving clients in shared decision-making, veterinarians can have a preference and state their preference, however, they need to ensure the client knows that their own thoughts, opinions, and beliefs are equally or more important than the veterinarian’s. Participating pet owners’ expectations of developing a partnership with their veterinarian included an expectation that veterinarians treat an owner’s knowledge, beliefs and expectations with equal importance. Further supporting the use of shared decision-making are pet owners’ expectations of having information tailored to their preferences, which is necessary to ensure that the client feels adequately informed to provide input in decisions. Although previous studies have reported that veterinarians may benefit from efforts to increase collaboration with their clients [1,3,34], shared decision-making has been minimally studied in veterinary medicine and is an area which requires additional research in the veterinary field.

Veterinarian-specific barriers to information exchange and decision-making reported in the current study remain consistent with general communication barriers reported previously by practicing veterinarians [11]. Time constraints, involvement of multiple clients in the care of a pet and language barriers have been consistently recognized as barriers for veterinarians [11,20]. Interestingly, veterinarians discussed time pressures in relation to their ability to follow-up with clients and the effect that visibly being rushed may have on clients’ perceptions, yet previous focus groups with veterinarians have described time pressure affecting their ability to communicate sufficient information within an appointment [11,20]. One study [36] found that significantly more veterinarians appeared rushed during problem appointments compared to wellness appointments, suggesting that allocating appointment time based on appointment-type may benefit veterinarians by reducing impediments on account of time constraints. Further, understanding factors that influence pace, content and duration of an appointment may increase client satisfaction [37], as this could enhance tailoring of scheduled appointment times.

This study used focus group methodology to collect and analyze the perspectives of pet owners and veterinarians. Guidance on the ideal number of focus groups varies widely. Guest [38] suggests that 80% of themes are identified within the first three focus groups, while 90% of themes can be identified within three to six focus groups. The final pet owner and veterinarian focus groups in the present study confirmed that data saturation [39] had been reached, as no new codes were identified. While ensuring validity in qualitative research has been debated [40,41], two primary techniques were used to enhance the validity of the current analysis. Member checking [16] was used to promote trustworthiness of the data, which involved reviewing main topics discussed and their meaning with participants at the end of each focus group. Peer debriefing [16] was also used to maintain validity of the data throughout the analysis process. In addition, potential bias due to dominant individuals in the focus groups was minimized by analyzing data across focus groups rather than relying on one focus group or one set of participants to define a theme or subtheme. The qualitative methodology and non-probability sampling methods used in the current study limits the ability to generalize findings to the general pet owner and veterinarian populations. While pet owners and veterinarians were recruited from similar sampling populations, most, if not all pet owner participants did not have an existing veterinarian-client relationship with participating veterinarians.

Veterinarians should consider how the current findings reflect the expectations of their own clients during their veterinarian-client-patient interactions. Additionally, veterinarians and veterinary educators ought to consider the similarities found between studies that have qualitatively explored pet owners’ expectations over the past two decades to gain insight on general expectations that continue to prevail [11–15,20], while reflecting on newly emerging themes. Veterinarians may want to integrate specific communication skills that could address pet owner expectations acknowledged herein. Continuing education programs and practical guidebooks [42,43] have been developed to enhance practicing veterinarians’ communication skills and are encouraged for practicing veterinarians who wish to enhance their communication skills.

## Conclusions

The results of the present study offer practicing veterinarians and veterinary educators, insight into the concordance between pet owners’ and veterinarians’ perceptions of information exchange and clinical decision-making. While previous research found that there was uncertainty around whether veterinarians or pet owners were driving the expectation of partnership [11], it is evident from the current study that pet owners drive this expectation. Though many pet owner expectations in relation to communication of information have continued to prevail over the past decade, additional expectations related to the increase in accessibility of information have emerged in the present study compared to previous qualitative studies [11–13,15]. The veterinary field should consider developing best practices for veterinarian-client communication regarding internet use, as the internet is widely used by pet owners to access information and offers an opportunity for veterinarians to assist pet owners in accessing reliable resources. In addition, the current study identified implications of providing recommendations to pet owners without offering additional options. Veterinarians who use a more paternalistic approach to communication may want to consider how this affects owners’ perceptions. Pet owners’ expectations for tailored communication of information based on their needs and being informed upfront about their options, align with the concept of shared decision-making and support the exploration of shared decision-making in companion animal practice. Best practices should be explored for veterinarians to participate in shared decision-making in an efficient and economical manner and be assessed for its effectiveness in client satisfaction and health outcomes in veterinary medicine.
